# The Impact of Human Conflict on the Genetics of *Mastomys natalensis* and Lassa Virus in West Africa

**DOI:** 10.1371/journal.pone.0037068

**Published:** 2012-05-15

**Authors:** Aude Lalis, Raphaël Leblois, Emilie Lecompte, Christiane Denys, Jan ter Meulen, Thierry Wirth

**Affiliations:** 1 Département Systématique et Evolution, Muséum National d'Histoire Naturelle, Paris, France; 2 Centre de Biologie et Gestion des Populations, Montferrier-sur-Lez, France; 3 Laboratoire Evolution et Diversité Biologique, Toulouse, France; 4 Institute of Virology, Philipps University, Marburg, Germany; 5 Laboratoire de Biologie intégrative des populations, Ecole Pratique des Hautes Etudes, Paris, France; Louisiana State University, United States of America

## Abstract

Environmental changes have been shown to play an important role in the emergence of new human diseases of zoonotic origin. The contribution of social factors to their spread, especially conflicts followed by mass movement of populations, has not been extensively investigated. Here we reveal the effects of civil war on the phylogeography of a zoonotic emerging infectious disease by concomitantly studying the population structure, evolution and demography of Lassa virus and its natural reservoir, the rodent *Mastomys natalensis*, in Guinea, West Africa. Analysis of nucleoprotein gene sequences enabled us to reconstruct the evolutionary history of Lassa virus, which appeared 750 to 900 years ago in Nigeria and only recently spread across western Africa (170 years ago). Bayesian demographic inferences revealed that both the host and the virus populations have gone recently through severe genetic bottlenecks. The timing of these events matches civil war-related mass movements of refugees and accompanying environmental degradation. Forest and habitat destruction and human predation of the natural reservoir are likely explanations for the sharp decline observed in the rodent populations, the consequent virus population decline, and the coincident increased incidence of Lassa fever in these regions. Interestingly, we were also able to detect a similar pattern in Nigeria coinciding with the Biafra war. Our findings show that anthropogenic factors may profoundly impact the population genetics of a virus and its reservoir within the context of an emerging infectious disease.

## Introduction

Lassa virus (LASV), an arenavirus and biosafety level 4 agent, is endemic in West Africa, where it causes up to 100,000 cases of Lassa fever (LF) per year and occurs sporadically in outbreaks with high mortality [1]. Humans are thought to become infected through contact with infected rodent excreta, urine, tissues, or blood [2], [3], [4], moreover, the contribution of bushmeat to the disease transmission should not be neglected [5]. LF was first described in 1969 [6] and is considered to be an emerging infectious disease (EID). The natural reservoir of LASV is the multimammate rat *Mastomys natalensis*, which is found over a wide geographic range encompassing much of sub-Saharan Africa and which lives in a peridomestic fashion in and around human dwellings [7]. For unknown reasons, LF is confined mainly to Nigeria, Sierra Leone, Liberia, Mali and Guinea [8] and LASV infected rodents are detected only focally in these countries. An explanation for the sporadic and focal endemicity of LASV infection in West Africa would have far reaching implications with respect to predicting and controlling possible outbreaks of the disease. While geographic distribution of LF seem to correlate with certain climatic conditions, particularly high rainfall [8], host population structure and genetic differences have not been extensively investigated and might explain the disease focality. To this end, we studied the population structure, evolution and demography of both LASV and its natural reservoir in Guinea, West Africa, covering also the borders with the other Mano River countries (Sierra Leone and Liberia). This region has been engulfed in civil war and political instability for the last two decades. More than 500,000 refugees have fled from Sierra Leone and Liberia to neighbouring Guinea, ranking this small country number one in Africa in terms of its refugee contingent [9].

## Results

**Figure 1:**
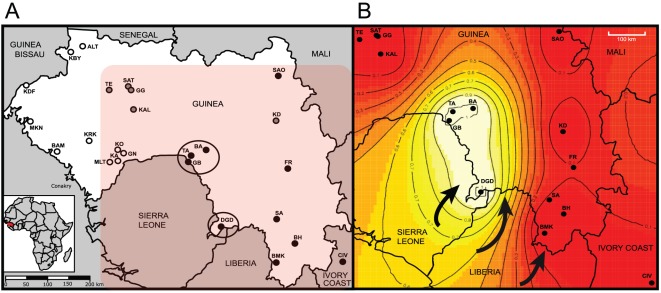
A) Mano River region map with the location of 25 trapping sites. Sites where only *Mastomys erythroleucus* or *M. natalensis* were trapped are shown in white and black dots respectively; sites where both species were captured are colored in gray. Regions where Lassa virus-positive rodents were caught are marked with large black circles (Districts of Faranah: villages of Gbetlaya (GB), Bantou (BA), Tanganya (TA) and Guéckédou: Denguedou (DGD). The reddish rectangular inset corresponds to the area represented in part B. B) Maps of Geneland spatial assignments to clusters for *K* = 2. The highest membership values are in light yellow and the isolines (grey curves) illustrate the spatial changes in assignment values. The plot is based on the highest-probability run for *K* = 2 (the same split and similar posterior probabilities were obtained for all 20 replicates).

To characterize the genetic variation within and between the rodent populations, we genotyped 9 unlinked microsatellite loci in 656 *M. natalensis* individuals. This sampling covered most of the Guinea habitats and geographic ranges (Figure 1A and Table S1). We applied two complementary Bayesian clustering algorithms, namely Structure 2.1 [10] and Geneland 3.1.4 [11] to infer rodent population structure and to probabilistically assign individuals to populations or clusters based on individual multilocus genotypes. The second algorithm also included the spatial origins of the rodents in the analyses. Using a hierarchical approach, we investigated the first split (*K* = 2) that could be detected using the spatial model for landscape genetics implemented in Geneland (Figure 1B). Interestingly, all samples collected close to the border with Sierra Leone clustered together. This binary split provided the most stable genetic partition, whereas higher splits were hardly converging. Moreover, these Sierra Leone border populations (Tanganya, Bantou, Gbetaya and Denguedou) were the only ones found to be LASV positive by RT-PCR [7], suggesting a shared ancestry or epidemiological link.

We evaluated the probabilities of not observing Lassa positive rodents in the other samples (the ones assigned as Lassa virus negative). To do so, we extrapolated a prevalence from the Lassa positive samples P = (13+33+4+19)/(97+170+39+148) = 0.1520. This was our upper bound and we took a fairly lower prevalence of P = 0.02 as the lower bound. A binomial law was implemented to calculate the probabilities in each population of not observing any infected rodent. If the incidence is the same as the one we observed, the probability to fail to detect Lassa positive rodents in the “negative” populations with more than 14 rodents is <0.10. The probability to fail to find positive rodent in all “negative” samples is <1×10^−14^. The probability to fail to find positive rodent in all “negative” samples using the lower bound value is <0.05. Therefore, we believe that we have a fairly good global picture of the situation in the field, though we could not exclude the presence of “false” negatives in the populations with few individuals, but this is obvious without any statistics.

**Figure 2:**
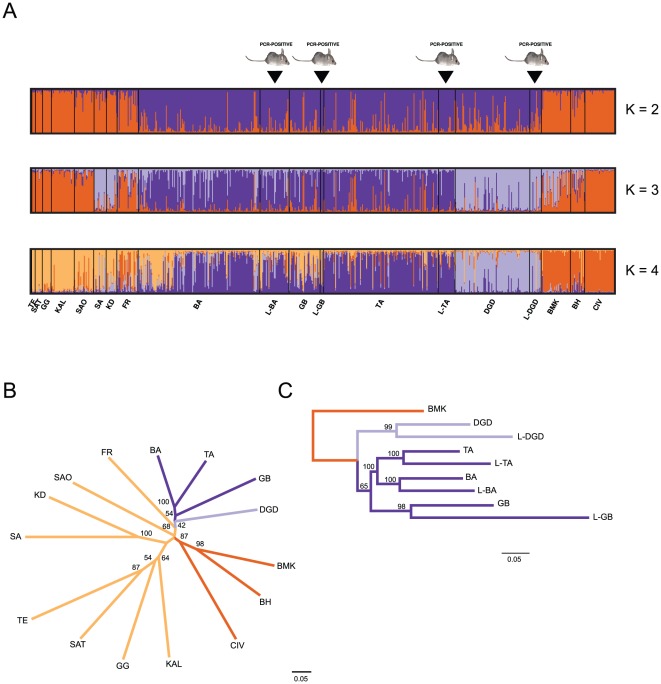
A) *M. natalensis* populations clustering based on Structure Bayesian inference (*K* = 2 to 4); Burn-in period = 150,000; MCMC repeat length = 200,000). Each color represents one assumed population cluster *K*. Multiple colored bars display an individual's estimated membership proportion in more than one population (q), i.e. the admixture level. The labels correspond to the sampling location indicated in table S1. It should be noted that PCR-positive and PCR-negative rats collected in a same location belong to the same source population according to structure. B) Neighbor-joining (NJ) phenogram summarizing Cavalli-Sforza & Edwards' (1967) *D_CE_* chord distances among 15 populations of *M. natalensis* collected in Guinea. Values on the nodes represent the percentage of bootstrap replicates over individuals (n = 100). Branch lengths are proportional to the genetic distance between the different populations and the scale bar represents a distance *D_CE_* of 0.05. Population codes are defined according to Table S1. C) The same phylogenetic reconstruction as shown in (B) but with a partition of the LASV positive and negative rats into sub-populations. The Bamakama sample was used as an out-group.

Structure 2.1 provided consistent results over 20 replicated runs and the probability of the data (LnPr(X|K)) increased from *K* = 1 to *K* = 15 although with a clear tendency to reach a plateau (Figure S1). According to the Evanno test [12], *K* = 4 is the most likely scenario (Figure 2A), followed by *K* = 2, which has nearly the same Δ*K* value than for *K* = 7 and *K* = 11 but with less parameters. Structure results for *K* = 2 were fully congruent with the Geneland bipartition. At *K* = 4 the clustering reflects geographic and habitat jumps; the first cluster is composed of the sole Denguedou population and is followed by a central, a south-eastern and a western cluster. This genetic partitioning was also supported by phylogenetic reconstructions using the Cavalli-Sforza and Edward's chord distance (Figure 2B). This trend was also supported by the fact that all rodent LASV-free populations had significantly lower *F_is_* values than the populations harbouring the virus (t-Test, *P*<0.03).

An important issue of the study was to determine whether the LASV-PCR positive *M. natalensis* constitute a monophyletic group within the larger *M. natalensis* population, or whether LASV infection is distributed across the *M. natalensis* population without clear evidence of an association between particular rodent genetic characteristics and LASV reservoir status. According to Structure (Figure 2A) and phylogenetic bootstrapping (Figure 2C) the answer is clear; the LASV positive rodents share the vast majority of their ancestry with their source population, and the bootstraps values of the LASV positive and LASV negative sub-samples from the same source population were always ≥98%. Therefore the RT-PCR positive rodents from one location do not differ in their allelic frequencies from the RT-PCR negative rodents from the same station. However, there are some genetic features that do distinguish these sub-samples (Figure S2); in all comparisons, the inbreeding coefficient (*F_is_*) was significantly higher in the LASV positive sub-sample; moreover, the observed heterozygosities (*H_o_*) and allelic richness (*A*) were also significantly lower, with one exception. However, relatedness analyses using the Colony 2.0.0.1 [13] algorithm did not indicate an increase in the proportion of full- and half-sibs in the LASV positive rodents when compared to the healthy individuals from the same population. The four pairwise comparisons concerning the full-sibs were non-significant and only one comparison out of four was statistically significant (*P* = 0.02) at the half-sib level (Table S2). We then tested by multivariate analysis of variance (MANOVA) the possible effects of LASV infection, locality, sampling year and season on the different genetic parameters. LASV was the only variable, which was significantly correlated with *F_IS_*, *H_o_* and *A* (Table 1).

**Table 1:** Summary statistics of Multivariate Analysis of Variance (MANOVA): The mixed – effects (Lassa Virus infection, Locality, Year and Season) on three parameters of genetic diversity (Inbreeding coefficient, *F_is_*, Observed heterozygosity, *H_o_* and Allelic richness, *A)* of four *Mastomys natalensis* populations where both LASV-PCR negative and LASV-PCR positive individuals coexisted.

	*F_is_* (coefficient of inbreeding)			*H_o_* (observed heterozygosity)		*A* (allelic richness)	
Source of variation	Df	*F*-value	*P*-value	*F*-value	*P*-value	*F*-value	*P*-value
Lassa Virus Infection	1	18.421	<0.001***	21.014	<0.05*	17.256	<0.001***
Locality (Loc)	3	0.856	0.623	0.652	0.780	0.469	0.848
Year	2	0.987	0.252	0.451	0.213	0.486	0.549
Season	1	0.396	0.478	1.002	0.512	0.899	0.148
Interaction LASV×Loc.	3	0.965	0.854	0.899	0.746	1.231	0.785
Interaction LASV×Year	2	0.423	0.790	0.451	0.390	1.478	0.328
Interaction Loc×Year	3	0.462	12.458	0.841	11.485	0.863	15.745

(*Significant at the 0.05 level, *** significant at the 0.001 level).

Coalescent-based inference methods can also provide interesting insights into a population's past demography [14]. The Msvar algorithm indicated that all *M. natalensis* populations had undergone a severe and recent population decline (Table S3). Inferred current effective population sizes were extremely small (between 2.9 and 8.5 individuals), whereas ancestral effective population sizes were estimated to be between 58,000 and 91,000 individuals. According to Msvar this decline occurred ten to twenty years ago and simultaneously in all sampled populations.

**Figure 3:**
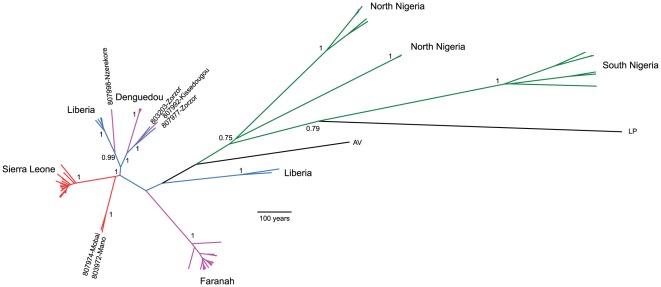
Maximum clade credibility tree representing the genealogy of the LASV strains by analyzing 627 nucleotides of the nucleoprotein gene using a Bayesian method. The tree has a proportional relationship between branch length and time. The main lineages are highlighted. The numbers represent posterior probabilities of the most important internal nodes. Color code: Green: Nigeria; blue: Liberia; Red: Sierra Leone and Pink: Guinea. Strains abbreviation and characteristics are described in Tables S4.

**Figure 4:**
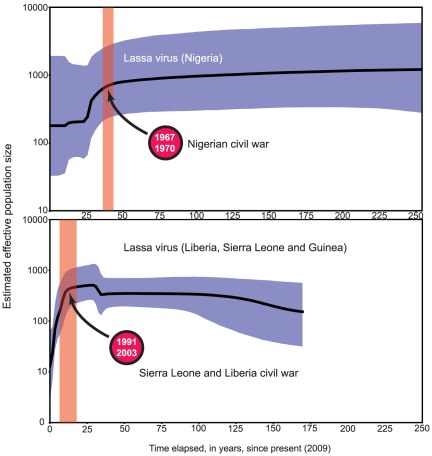
Beast Bayesian skyline plots obtained by analyzing LASV nucleoprotein sequences from 18 Nigerian and 72 Mano River region strains sampled at different times. The black solid line represents the median and the grey area the 95% HPD of the *N_e_*(*t*) estimates. The orange columns highlight civil war peaks in the concerned regions and illustrate the concordance between civil war, virus population decline and viral diversity loss.

LASV infection was detected in 69 out of 656 rodents (10.5%) by RT-PCR. Because *M. natalensis* is the only known reservoir for LASV and since directly transmitted pathogens can provide information on the temporal and spatial characteristics of the host-to-host contact [15], we analysed the LASV phylogeography by partial sequencing of the nucleoprotein gene (NP) from viral strains circulating in the natural reservoir during a 3-year period in Guinea. The choice of this genetic marker was also guided by the availability of a large dataset of isolates collected from 1969 to the present covering the complete LASV distribution range in West Africa [16], which was also included in the analyses (Table S4). Before applying coalescent theory to assess the demographic history of LASV, we first checked the sequences for recombination events which would strongly interfere with the model assumptions. No recombination events were detected using any of the six approaches for recombination detection implemented in the Rdp3 package [17], and therefore the NP data set can be used in Beast 1.4 without modification [18]. Figure 3 depicts an unrooted maximum clade credibility tree, with the posterior probabilities for the branches separating the main lineages. The topology of the tree confirms the presence of two major clades corresponding to two geographically separate endemic areas; the Mano River region (MRR) in the west and Nigeria in the east [16]. If we consider the first described strain of LASV (LP) as the root of the tree, the topology strongly argues for an eastern origin for this haemorrhagic fever. The same topology is observed when the tree is rooted with the Mobala virus (data not shown). Furthermore, the genetic diversity is higher in Nigeria (π = 0.123) when compared to Western Africa (π = 0.085). Both the strict and relaxed clock (RC) models were implemented using the different demographic models, but in each case the RC exponential model was the best supported. The estimated evolutionary rate (Table 2) was 1.6×10^−3^ substitutions/site/year (95% Highest Posterior Density (HPD), 8.1×10^−4^–2.5×10^−3^) for the MRR lineage, which falls within the reported range of negative-sense RNA viruses [19]. We also attempted to calculate the time-lapse backup to the most recent common ancestors (TMRCA) of the sampled viruses, as well as the age of the different lineages and viral populations sampled where human LF cases have occurred. For the global LASV sample, the root was dated to 757 years ago (95% HPD: 504–1053, data not shown). However, given that two LASV clades are distinct (MRR and Nigeria) and may have contrasting demographic histories, we decided to run independent Bayesian analyses on each of them (Table 2). The Bayes factors (BF) analysis showed that the Bayesian skyline plots fitted the data better than the constant model at a decisive level of evidence (log BF>3). The overall MRR TMRCA was estimated to be 163 years ago (95% HPD: 64–317), whilst we obtained estimates of 42 years ago for the Faranah district (95% HPD: 8–78) and only 8 years in Denguedou (95% HPD: 6–13). It should be noted that the LASV demogenetic parameters in Nigeria and West Africa are contrasted. Whilst the Nigerian viral population appears to have remained constant during the last 250 years, it underwent a mild bottleneck some 40 years ago before stabilizing over the last 25 years. In contrast, the MRR LASV population was stable until very recently but dramatically declined (by a factor 500) within the last 15 years (Figure 4). Interestingly, running the Bayesian algorithm on the Nigerian strains alone resulted in a higher estimate of the TMRCA, 911 years (95% HPD: 47–2,798), although with a much larger confidence interval as a consequence of the smaller sample size.

## Discussion

In order to build a possible evolutionary and biogeographic scenario for this haemorrhagic fever virus, we then combined the genetic data gathered from *M. natalensis* and LASV. The virus phylogenetic reconstruction points toward a Nigerian origin of LASV, since the TMRCA estimate was nearly identical for the Nigerian populations and the global sample, i.e. about 750 to 900 years ago. The rather late arrival of LASV in Western Africa, 150–250 years ago during the colonial period, is surprising as it raises the question of whether human activities have played a role in this event. A further currently unexplained observation is the near absence of LF cases from Côte d'Ivoire, Ghana, Togo and Benin, possibly due to lower rainfall and milder temperatures [8]. Neither LASV nor LF have been reported in these countries to date. However, other arenaviruses are almost certainly present. For example in Côte d'Ivoire two novel virus species were recently detected in 6/1300 rodents and LASV antibodies have been detected in humans (S. Gunther, pers. comm.). Lassa fever is considered to be endemic in Sierra Leone and has occurred in outbreaks with high mortality rates between 1971 and 2000 [8]. During the civil war in Sierra Leone from 1991 to 2003 there was a massive influx of refugees into Guinea along its southern border and our analysis shows that this coincided with both a sharp decrease in the size and genetic diversity of the populations of *M. natalensis* and LASV (Figure 4 and Table S3). We propose two non-exclusive explanations for this observation, both linked to human activities. One is the large-scale deforestation that occurred in the refugee areas. Some of this deforestation has been documented for example in a small strip of land called the “Parrot's Beak” (location of Denguedou) by the United Nations Environment Program (http://unepatlas.blogspot.com/2008/06/guinea-refugee-camps.html). It is clear that ecological perturbations will have had a severe impact on the population structure of *M. natalenis*. The second explanation may be that in the forest regions of Guinea and Sierra Leone small animals including rodents are often hunted as supplementary protein sources, a practice which has been shown to carry a risk of LASV infection [5].

The situation is better described in Equatorial Guinea, where rodents were fond to represent nearly one third of the meat at markets [20], [21]. With large numbers of refugees in search of food this may have resulted in additional pressure on the rodent populations. This hypothesis is difficult to prove in the field [22]. However, Phillip Cullison Bonner (MD MSc) who worked in this area gave us the following information: “In all of the camps, subsistence hunting of rodents did occur although not everyone participated in this behavior. In one camp in particular, an NGO had placed a ‘bounty’ for rats killed and collected by the camp's inhabitants in order to control the Lassa problem. These killed rodents were buried at one site at the edge of a camp and refugees told me they later went back to dig them up to eat them saying that it was ‘wasted food’ if they did not dig them up. In our spatial analysis we noted a significant cluster of Lassa cases near the rodent burial site of this camp, though we have no means to show if this is coincidental or causal to the collection activity”.

Moreover, our population genetics analyses indicate that the rodent populations along the borders with Sierra Leone (i.e. Denguedou and Faranah) clearly belong to a distinct evolutionary lineage when compared with the other Guinean populations (Figure 1B and Figure 2AB). A likely scenario is that a concomitant migration of the refugee populations from Sierra Leone and Liberia with their peridomestic “resident” rodent populations may have occurred, and that this may explain the recently observed LF activity in the region in the years 2003 and 2005. Numerous cases of simultaneous colonization of human commensal species during human migrations have been reported [23], [24]. For example, the story of the peopling of the Pacific by the Polynesians was unravelled through Pacific rats mtDNA phylogenies [25]. This scenario is supported by the significantly higher *F_i_*_s_ values observed in the LASV positive populations (Figure S3), a classical founder effect seen in populations that have arisen from a small initial population.

We also tested our hypothesis concerning the putative link between war area and occurrence of cases as well as refugee camps and outbreaks. Using spatial randomization procedures (Figure S4) and home made R scripts we were able to show that outbreaks localities tend to be closer to refugee areas (Figure S5) than expected by chance alone, though being marginally significant (*P* = 0.055). However, this statistics was no more significant when comparing occurence of cases and conflict areas (*P* = 0.479). These results support our hypothesis that refugee camps are potentially important sites of LASV transmission.

Intriguingly, the analysis of the LASV NP sequences shows that the LASV population in Nigeria collapsed during the Nigerian civil war (1967–1970) and only stabilized during the last 25 years (Figure 3), mirroring the situation observed in Guinea at the present time.

This is, to our knowledge the first report of an impact of conflict situations on the phylogeography and demography of a virus. Previously, conflict situations have been found to impact the incidence of EIDs through different mechanisms, including population displacement, changes in environmental conditions, a breakdown in infection control, the disruption of disease control programs and the collapse of health systems and early warning and response systems [9], [26]. These factors undoubtedly all operate in conflict situations in Lassa endemic areas and have led to an increase of LF cases in local hospitals and among international aid workers [9]. However, the disruptive impact of conflict situations on the virus-reservoir relationship of LASV and the resulting genetic changes of the virus will have effects that may manifest themselves only over long periods of time. Theoretically, they could result both in an increase or decrease of LF cases, depending on the properties of the LASV strains selected and how they re-establish themselves in the rodent population.

An intriguing result of our study was the observed consanguinity among the LASV positive rodents. It has previously been reported that LASV infection in *M. natalensis* was accompanied by inflammatory lesions and that virus-positive animals were smaller in weight and shorter in length [27]. These observations suggest that LASV positive rodents might be less fit than the LASV negative ones and that ultimately sexual selection might drive those individuals to inbreeding. Moreover, a lowered fitness will likely result in more frequent encounters of rodents with humans and thus increased LASV transmission.

In summary, we propose that the focal occurrence of LF in West Africa and its status as an EID are not linked to genetic variation of the rodent host, but due to human perturbation of the virus-host relationship [28]. This could take the form of transportation of infected rodents across large distances (e.g. on ships or trucks), which then would establish new foci of LASV transmission in the local *M. natalensis* population, provided permissive climatic conditions are present [8]. Ecological change brought about by large population movements due to conflict situations will have a major impact on both the size and genetic variability of the local rodent and the virus population, which will result in unpredictable long-term effects on the epidemiology of LF. Though we consider them less unlikely, we cannot rule out alternative explanations: social, political, and ecological processes were ongoing over the time period explored, including some influences that, no doubt, are still unrecognized. Taken together, our findings reinforce the importance of rodent control measures not only to reduce the risk of LF in established foci, but also to prevent the spread of LASV to susceptible regions in Africa.

## Materials and Methods

### Ethics Statement

Animals were live-trapped and handled under the guidelines of the American Society of Mammalogists (ASM; http://www.mammalogy.org/committees/index.asp; Animal Care and Use Committee, 1998). The protocol was approved by the Ministry of Public Health, Republic of Guinea (permission no. 2003/PFHG/05/GUI). Trapped *Mastomys* were handled according to standard procedures for BSL3 work in the field (Federal Guidelines for Field Work, CDC 1997). The animals were killed by a lethal dose of isofluorane.

### Field sampling

In a survey of rodent borne hemorrhagic fever viruses, 957 *Mastomys* sp. were trapped in the Republic of Guinea and Côte d'Ivoire from 2002 to 2006. The animals were caught in 24 different Guinean study sites, which were each rural villages with a human population <1,000 and in one site in Côte d'Ivoire (Figure 1 and Table S1). The sampling effort was identical in all localities, with the exception of Tanganya and Bantou, where the trapping sessions were extended for temporal surveys. The variation in the number of *Mastomys* trapped at each site is the result of regional abundance differences in Guinea. For example, a geographical survey of Guinean rodents from 2002 to 2007 showed that *M. natalensis* was absent from Guinea Maritime and highly abundant in Forest Guinea [29]. Rodents were captured using Sherman traps (Sherman LFA live trap, H.B. Sherman Traps, Inc., Tallahassee, FL). Trapped *Mastomys* were handled according to standard procedures for BSL3 work in the field.

Cytogenetic analyses, PCR and DNA sequencing of cytochrome *b* were conducted for accurate identification of the species *M. natalensis* and *M. erythroleucus* since *M. natalensis* has been likely identified as the only host for LASV in Guinea [7]. Currently, two sampling areas in Guinea are at risk for the spread of Lassa virus: the prefecture of Faranah (TA, BA and GB) and the area of Denguedou (DGD), located near the region of Kenema, hearth of the hemorrhagic fever in Sierra Leone where epidemics are common [7] (see Figure 1).

**Table 2:** Beast Bayesian analyses for the 90 Lassa virus strains under different coalescent tree priors: constant population size, exponential growth, and skyline plots.

	Constant size			Exponential growth			Skyline		
	Lower	Mean	Upper	Lower	Mean	Upper	Lower	Mean	Upper
Age estimates (yr B.P.) Nigeria									
Root height	59	686	1,878	51	289	689	74	911	2,798
Marginal likelihood		−3529.3			−3527.3			−3526.0	
(substitutions/site/year)	1.8 10^−5^	3.3 10^−3^	7.4 10^−3^	4.1 10^−4^	4.9 10^−3^	1.0 10^−2^	5.5 10^−5^	1.9 10^−3^	4.7 10^−3^
Age estimates (yr B.P.) MRR									
Root height	90	250	501	88	221	416	64	163	317
Denguedou	8	17	29	7	16	28	6	8	13
Faranah	23	46	85	23	41	73	8	42	78
Sierra Leone	54	95	154	54	94	151	49	78	122
Marginal likelihood		−4923.8			−4922.7			−4920.5	
(substitutions/site/year)	6.3 10^−4^	1.3 10^−3^	2.0 10^−3^	7.4 10^−4^	1.4 10^−3^	2.0 10^−3^	8.1 10^−4^	1.6 10^−3^	2.5 10^−3^

Lower and upper estimates correspond to the 95% HPD intervals of the posterior probabilities.

### Rodent Microsatellite Genotyping

Total DNA was extracted from liver and spleen preserved in 70% ethanol using the DNeasy Tissue Kit (Qiagen). Nine microsatellite markers were amplified in a single multiplex PCR reaction with the Qiagen Multiplex PCR kit following manufacturer's instructions. PCR products were run on an ABI PRISM 310 instrument for allele separation. Genotypes were scored using GeneMapper 3.0 (ABI, Foster City, CA). All alleles were checked manually and appearance of variants outside existing bins was triple-checked (i.e. two repeat amplifications and scoring steps took place).

### Statistical analyses

Genetic variation was estimated over all loci within each population from the observed (*H_O_*) and expected (*H_E_*) heterozygoties [30] using the program Genetix version 4.05.2 (Belkhir *et al.* 1996–2004). To compare the allelic richness (*A*) in our different populations and to estimate the expected number of alleles for a given sample size, we used the rarefaction procedure implemented in Fstat 2.9.3.2 [31].

Genotypic linkage disequilibria between all pairs of loci, conformation to the Hardy–Weinberg equilibrium in each population for each locus and across all loci, as well as genotypic differentiation between populations were all tested by exact tests using Markov chains algorithms implemented in Genepop version 4 [32]. For all analyses, corrections for multiple tests were performed using the false discovery rate approach [33]. Genepop4 was also used to estimate Wright's *F* statistics (*F_ST_*, *F_IS_* and *F_IT_*) calculated according to the method of Weir & Cockerham [32].

Analysis of molecular variance [34] (AMOVA) subdivides genetic diversity into hierarchical components and was performed using ARLEQUIN 3.1. The variance components included in this analysis were: (i) between natural geographic area (guineo-congolese forest zone, savannah forest transition zone, sahelo-sudanian savannahs zone); (ii) between localities; and (iii) within a population in a locality. We investigated genetic differentiation further using *F_ST_* estimates calculated between populations. The statistical significance of variance components and F_ST_ indices was evaluated by randomization procedures. All statistical analyses (Two-sample independent t test, Multivariate Analysis of Variance) were done with the software R v.2.5.0 (http://cran.r-project.org).

### Parentage and sibship inference

To evaluate the potential effect of sampling rodent families on different population genetic estimators, we used the software Colony 2.0.0.1 [13], [35]. This maximum-likelihood method searches for the partition of a sample of individuals into full- and half-sib clusters. The analysis was set to allow for both polyandry/polygyny and inbreeding [36]. The pair-likelihood scores option, with the “medium length of run” and “medium likelihood precision' was activated. The overall rate of genotyping error and mutations was 10% (5% null alleles and 5% other types of mutations and genotyping error). Only relationships supported by probabilities of at least 0.9 were assumed to be correct. Finally, we statistically evaluated the plausible association of elevated *F_is_* values in LASV positive rodents with increased sibship and familial transmission using Poptools v3.2.3 [37]. Since the number of non-carrier *M. natalensis* (*N_x_*) always outnumbered the LASV positive rodents (*n_x_*) within each population, pairwise assignment scores of size *n_x_*×*n_x_*/2 were drawn a thousand times at random from the *N_x_*×*N_x_*/2 pairs generated in the LASV negative rodents. Frequencies distributions were plotted and the fit of the LASV positive score within the 95% interval confidence evaluated.

### Inferring the rodent population structure

We ran the admixture model of Structure 2 [10] for 200,000 iterations of the Gibbs sampler after a burn-in of 150,000 iterations. The correlated allele frequency model was used with asymmetric admixture allowed. We applied Structure to the entire data with *K* varying from 1 to 15, with 20 runs for each *K* value. In our analysis, the likelihood increased with increasing values of *K*, but slowly reached a plateau. The number of contributing populations was statistically tested using the ad-hoc Evanno statistic Δ*K* [12]. This procedure is sensitive to pronounced changes in mean log likelihood values between successive *K* values and the degree of variance of any given mean. The graphic display of the Structure results was generated using Distruct (Rosenberg 2004).

We also ran the spatial model of Geneland 3.1.4 [11] with the Dirichlet model for allelic frequencies. We first performed a preliminary analysis with 10 runs of 1 000 000 iterations with a thinning of 500 and a burn-in of 50%, considering values for *K* from 2 to 15 with a starting value of 9, to infer the number of populations *K* maximizing the posterior probability of the data. Then longer runs (ten replicates, each) of 20 000 000 iterations with a thinning of 500 and burn-in of 50% were analyzed to precisely set the spatial limits of the different populations for *K* = 2 (first split) and *K* = 10 (the split with the highest likelihood). For all analyses, the uncertainty attached to spatial coordinates was set to 0.2 km and the maximum number of nuclei in the Poisson-Voronoi tessellation fixed at 1800 (roughly three times the number of analyzed individuals).

Finally, Cavalli-Sforza and Edwards' chord distance [38] was used to construct a phylogenetic tree using a neighbor-joining algorithm [39] implemented in Populations v1.2.30b (http://bioinformatics.org). Support for the tree nodes was assessed by bootstrapping over individuals (100 iterations).

### Coalescence, TMRCA and demography

In a first step, we used a Bayesian approach implemented in MsVar 1.3 [14] that assumes a stepwise mutation model and estimates the posterior probability distributions of the genealogical and demographic parameters of a sample using Markov chain Monte Carlo simulations based on microsatellite data. This method permits inferences of important biological parameters such as the time to the most recent common ancestor (TMRCA) of a given sample in years, the past and present effective population size and the latest demographic changes (decline, constant population size or expansion). For this analysis, we focused on populations where at least 24 rodents were available, in order to get a reliable cover of the TMRCA and to avoid small sample size artifacts. The version 1.3 of MsVar provides separate estimates of the actual population size (*N_0_*), the ancestral population size (*N_1_*), the mutation rate per locus per generation (*μ*) and the time in year that have elapsed since the decline or expansion began (*T*). It relies upon a hierarchical model where demographic and mutational parameters are allowed to vary among loci and are described by a set of parameters Θ = {*N_0_*, *N_1_*, *T*, *μ*} whose prior distributions depend upon hyper-priors (see [40] for further details). We used relatively uninformative flat log-normal priors for all parameters with standard deviations of 2. A prior mean mutation rate of 10^−4^ was considered, based on former mouse and rat gene mapping experiments [41], [42], and prior means of 10^2^ were considered for time and population size parameters. The analyses were performed assuming an exponential demographic change. Three different chains of 1.6 10^9^ iterations and a thinning of 20 000 were run for each analysis to confirm the convergence of the results. Contraction signatures assessed with a burn in of 50% were robust and were confirmed with additional runs where an expansion was assumed as a prior.

### Individual Lassa virus gene dataset

All sequences were aligned using the clustalx 2.0 program [43] and sites with gaps were removed. Total RNA was extracted from rodent blood preserved in liquid nitrogen by using the Blood RNA kit (Peqlab, Erlangen, Germany). Extracted RNA was then screened for Lassa virus by a reverse transcription/polymerase chain reaction (RT/PCR), targeting the highly conserved polymerase (L) – gene of Lassa virus. For the LV analyses, we combined 90 individual sequences from two nucleoprotein gene data sets (627 bp). They included single genome amplified sequences from 46 human and 12 rodent Lassa virus strains covering the geographical distribution range of Lassa virus as known in 2006 [16] and 32 rodent strains from Guinea collected from 2002 to 2005 [7], as well as the reference strains (see table S4 for details).

### Detection of recombination in LASV

Putative recombinant sequences were identified using five independent recombination detection programs with the Rdp3 package [17]: rdp, geneconv, Maxchi, Bootscan, 3seq and Ldhat. We used the default detection thresholds for all analyses and removed putative recombinant sequences identified by at least two of these programs. This approach is rather conservative and ensures that our results have a high likelihood of being unaffected by recombination; however though being highly selective there was no evidence for phylogenetic incongruence indicative of recombination in this data set.

### Phylogenetic inferences and coalescent analyses

Phylogenetic relationships were reconstructed using the maximum likelihood (ML) approach implemented in Phyml 3.412 [44]. The robustness of the ML tree topology was assessed with bootstrapping analyses of 1,000 pseudo-replicated datasets. A generalized time reversible (GTR) substitution model [45] with gamma distributed rate heterogeneity [46] and a proportion of invariable sites was selected based on Akaike's information criterion using jmodeltest [47]. Phylogenies were rooted with the prototype LP strain collected in 1969 from the village of Lassa, in northeastern Nigeria.

The specific rate of evolution for the NP was estimated from the “serially-sampled” Lassa virus strains with known dates of sampling (range 1969 to 2005, n = 90). Evolutionary rates were obtained using the Bayesian MCMC approach implemented in beast 1.4 [18], [48]. An uncorrelated lognormal relaxed molecular clock was chosen, which assumes no *a priori* correlation between a lineage's rate of evolution and that of its ancestor. During analysis, evolutionary rates and tree topologies were analyzed using the GTR and Hasegawa-Kishino-Yano [49] (HKY) substitution models with gamma distributed among-site rate variation with four rate categories (Γ_4_). Constant-sized, logistic, exponentially growing coalescent models were used. We also considered the Bayesian skyline plot model [50], based on a general, non-parametric prior that enforces no particular demographic history. We used a piecewise linear skyline model with 10 groups and we then compared the marginal likelihood for each model using Bayes factors estimated in tracer 1.4. Bayes factors represent the ratio of the marginal likelihood of the models being compared. A ratio between 3 and 10 indicate a moderate support that one model is a better fit to the data than another, whereas values larger than 10 indicate strong support. For each analysis, two independent runs of 50 million steps were performed. Examination of the MCMC samples with tracer 1.4 indicated convergence and adequate mixing of the Markov chains, with estimated sample sizes in the hundreds or thousands. The first 10% of each chain were discarded as burn-in. We summarized the MCMC samples using the maximum clade credibility topology found with treeannotator 1.4 [18], with branch length depicted in years (median of those branches that were present in at least 50% of the sampled trees; Figure 2). The Bayesian skyline plot was reconstructed using the posterior tree sample and tracer 1.4.

### Spatial analyses and correlation between outbreaks and war localities and refugee camps

We tested the hypothesis concerning a putative link between war area and occurrence of cases as well as refugee camps and outbreaks using home made scripts written in R (Appendix S1). We focused on a large area that comprises Sierra Leone, Liberia, Guinea, and the western part of Ivory Coast (Figure S4). To correlate occurrence of cases with the presence of refugee camps and conflict areas, we implemented a first algorithm in order to calculate the distances between outbreak localities and the closest refugee camp or the closest conflict areas. The human outbreak localities (N = 73) were chosen according to Fichet-Calvet et al. (2009). Then, another algorithm was written in order to generate randomly the same number of points on the study area map and calculate the distance of those points to the refugee camps or conflict areas. This model takes into account the population density of the area and each generated random point is accepted with a probability proportional to the population density at this location deduced from SEDAC GPW maps (http://sedac.ciesin.columbia.edu/gpw/global.jsp). This density dependant approach avoids improbable affectation of random outbreaks in deserted areas and assumes that outbreaks of Lassa fever are more likely in regions with higher densities of population. We ran the algorithm 10, 000 times and compared the mean distances of each repetition to the mean distance of the real outbreaks. The scripts and analyses are described in the supplementary online material.

## Supporting Information

Figure S1 Estimated number of clusters and genetic structure inferred using structure version 2.2 [10]. Black diamond symbols indicate average log-likelihoods from 20 replicates for each assumed number of populations (*K*), and errors bars correspond to 1 s.d. White diamond symbols indicate values of the *ad-hoc* statistic Δ*K*, which is based on the rate of change of the log-likelihood as *K* is increased. Δ*K* tends to peak at the value of *K* that corresponds to the highest level of hierarchical substructure [12].
(EPS)

Figure S2 Inbreeding coefficient (*F_is_*), observed heterozygosity (*H_o_*) and allelic richness (*A*) of four *Mastomys natalensis* populations, where both LASV-PCR negative and LASV-PCR positive individuals coexisted. White, PCR negative sub-samples; light grey, PCR positive sub-samples. Boxplots of *F_is_*, *H_o_* and *A* are represented. N.S., non significant pairwise comparison at the 5% level; * *P*<0.05; ** *P*<0.01 and *** *P*<0.001. Abbreviations, BA = Bantou; GB = Gbetaya; TA = Tanganya; DGD = Denguedou and L stands for Lassa virus PCR positivity. The *P* values in all boxes charts were calculated using the two-sample independent *t* test.
(EPS)

Figure S3 Mean *F_is_* values calculated over LASV free and LASV positive *M. natalensis* populations. In order to evaluate the impact of the highly inbred LASV positive rodents within each population, we recalculated the *F_is_* removing those individuals from their source population. Please note that this correction did not affect the significance of the test. Therefore the rodent populations flanking the border display significantly higher inbreeding coefficients than their counterparts.
(EPS)

Figure S4 A) Synthetic map presenting the conflicts areas and years of occurrence in Guinea, Liberia, the Western part of Côte d'Ivoire and Sierra Leone, as well as the refugee camp sectors. B) Map showing the population density in the area of interest. The probability of choosing a point randomly in our re-sampling procedure was weighted by a density-dependant probability to take into account the number of inhabitants/km^2^.
(EPS)

Figure S5 A) Distribution of the mean distance to the closest conflict area obtained from 10,000 iterations. In each step 73 points were randomly generated (reminiscent to the number of human outbreaks presented in Fichet-Calvet et al. 2009) and mean virtual distances from each point to its closest conflict area were calculated. The thick line corresponds to the real data (*P* = 0.479). B) Distribution of the mean distance to the closest refugee camp obtained from 10,000 randomization procedures of 73 points (reminiscent to the number of human outbreaks presented in Fichet-Calvet et al. 2009). The line corresponds to the real data (*P* = 0.055).
(EPS)

Table S1 Dates and locations of animal-trapping sites in Guinea and Ivory Coast. The areas were chosen according to the results published in Lukashevich et al. (1993) [51] and Demby et al. (2001) [52]. They cover representative sites of the principal geographic regions of Guinea where human/rodent presence of Lassa virus was observed.
(DOC)

Table S2 Pairs of putative full-sibs and half-sibs identified within stations using Colony 2.0.0.1 [13]. *P* values in the first line correspond to the probabilities of the sibship assignments. *P* values from the two last columns were estimated from resampling procedures implemented in Poptools v3.2.3 [37] and indicate if the LASV positive rats sub-sample in each population are significantly different from the LASV negative *M. natalensis* sub-samples.
(DOC)

Table S3 Summary statistics for the *M. natalensis* demographic changes (populations with ≥24 individuals). The estimates were obtained with the algorithm Msvar 1.3. [14] and correspond to effective population sizes and not absolute population sizes. The 95% confidence intervals are provided in the brackets.
(DOC)

Table S4 Complete listing of the sequences included in the phylogenetic analysis of the viruses, as identified from databases, or determined in the course of this study.
(DOC)

Appendix S1 Scripts implemented for the spatial analyses.
(DOC)

## References

[1] McCormick JB, King IJ, Webb PA, Johnson KM, O'Sullivan R (1987). A case-control study of the clinical diagnosis and course of Lassa fever.. J Infect Dis.

[2] Monath TP, Newhouse VF, Kemp GE, Setzer HW, Cacciapuoti A (1974). Lassa virus isolation from *Mastomys natalensis* rodents during an epidemic in Sierra Leone.. Science.

[3] McCormick JB (1987). Epidemiology and control of Lassa fever.. Curr Top Microbiol Immunol.

[4] Stephenson EH, Larson EW, Dominik JW (1984). Effect of environmental factors on aerosol-induced Lassa virus infection.. J Med Virol.

[5] Ter Meulen J, Lukashevich I, Sidibe K, Inapogui A, Marx M (1996). Hunting of peridomestic rodents and consumption of their meat as possible risk factors for rodent-to-human transmission of Lassa virus in the Republic of Guinea.. Am J Trop Med Hyg.

[6] Frame JD, Baldwin JM, Jr, Gocke DJ, Troup JM (1970). Lassa fever, a new virus disease of man from West Africa. I. Clinical description and pathological findings.. Am J Trop Med Hyg.

[7] Lecompte E, Fichet-Calvet E, Daffis S, Koulemou K, Sylla O (2006). *Mastomys natalensis* and Lassa fever, West Africa.. Emerg Infect Dis.

[8] Fichet-Calvet E, Rogers DJ (2009). Risk maps of Lassa Fever in west Africa.. PLoS Negl Trop Dis.

[9] Gayer M, Legros D, Formenty P, Connolly MA (2007). Conflict and emerging infectious diseases.. Emerg Infect Dis.

[10] Pritchard JK, Stephens M, Donnelly P (2000). Inference of population structure using multilocus genotype data.. Genetics.

[11] Guillot G, Santos F, Estoup A (2008). Analysing georeferenced population genetics data with Geneland: a new algorithm to deal with null alleles and a friendly graphical user interface.. Bioinformatics.

[12] Evanno G, Regnaut S, Goudet J (2005). Detecting the number of clusters of individuals using the software STRUCTURE: a simulation study.. Mol Ecol.

[13] Jones OR, Wang J (2010). COLONY: a program for parentage and sibship inference from multilocus genotype data.. Mol Ecol Resour.

[14] Beaumont MA (1999). Detecting population expansion and decline using microsatellites.. Genetics.

[15] Biek R, Drummond AJ, Poss M (2006). A virus reveals population structure and recent demographic history of its carnivore host.. Science.

[16] Bowen MD, Rollin PE, Ksiazek TG, Hustad HL, Bausch DG (2000). Genetic diversity among Lassa virus strains.. J Virol.

[17] Martin DP, Williamson C, Posada D (2005). RDP2: recombination detection and analysis from sequence alignments.. Bioinformatics.

[18] Drummond AJ, Rambaut A (2007). BEAST: Bayesian evolutionary analysis by sampling trees.. BMC Evol Biol.

[19] Chare ER, Gould EA, Holmes EC (2003). Phylogenetic analysis reveals a low rate of homologous recombination in negative-sense RNA viruses.. J Gen Virol.

[20] Juste J, Fa JE, Perez del Val J, Castroviejo J (1995). Market dynamics of bushmeat species in equatorial Guinea.. Journal of Applied Ecology.

[21] Fa JE, Juste J, Perez del Val J, Castroviejo J (1995). Impact of market hunting on mammal species in Equatorial Guinea.. Conservation Biology.

[22] Bonner PC, Schmidt WP, Belmain SR, Oshin B, Baglole D (2007). Poor housing quality increases risk of rodent infestation and Lassa fever in refugee camps of Sierra Leone.. Am J Trop Med Hyg.

[23] Wirth T, Meyer A, Achtman M (2005). Deciphering host migrations and origins by means of their microbes.. Mol Ecol.

[24] Wirth T, Wang X, Linz B, Novick RP, Lum JK (2004). Distinguishing human ethnic groups by means of sequences from *Helicobacter pylori*: lessons from Ladakh.. Proc Natl Acad Sci U S A.

[25] Matisoo-Smith E, Roberts RM, Irwin GJ, Allen JS, Penny D (1998). Patterns of prehistoric human mobility in polynesia indicated by mtDNA from the Pacific rat.. Proc Natl Acad Sci U S A.

[26] Pike BL, Saylors KE, Fair JN, Lebreton M, Tamoufe U (2010). The origin and prevention of pandemics.. Clin Infect Dis.

[27] Demartini JC, Green DE, Monath TP (1975). Lassa virus infection in *Mastomys natalensis* in Sierra Leone. Gross and microscopic findings in infected and uninfected animals.. Bull World Health Organ.

[28] Jones KE, Patel NG, Levy MA, Storeygard A, Balk D (2008). Global trends in emerging infectious diseases.. Nature.

[29] Denys C, Lalis A, Aniskin V, Kourouma F, Soropogui B (2009). New data on the taxonomy and distribution of Rodentia (Mammalia) from the western and coastal regions of Guinea West Africa.. Italian Journal of Zoology.

[30] Nei M (1978). Estimation of Average Heterozygosity and Genetic Distance from a Small Number of Individuals.. Genetics.

[31] Goudet J (1995). FSTAT (Version 1.2): A computer program to calculate F-Statisitics.. The Journal of Heredity.

[32] Rousset F (2008). Genepop'007: a complete reimplementation of the Genepop software for Windows and Linux.. Mol Ecol Resources.

[33] Benjamini Y, Hochberg Y (1995). Controlling the false discovey rate – a practical and powerful approach to multiple testing.. J R Stat Soc Ser B.

[34] Excoffier L, Smouse PE, Quattro JM (1992). Analysis of molecular variance inferred from metric distances among DNA haplotypes: application to human mitochondrial DNA restriction data.. Genetics.

[35] Wang J (2004). Sibship reconstruction from genetic data with typing errors.. Genetics.

[36] Wang J, Santure AW (2009). Parentage and sibship inference from multilocus genotype data under polygamy.. Genetics.

[37] Hood GM (2010). http://www.poptools.org.

[38] Cavalli-Sforza LL, Edwards AW (1967). Phylogenetic analysis. Models and estimation procedures.. Am J Hum Genet.

[39] Saitou N, Nei M (1987). The neighbor-joining method: a new method for reconstructing phylogenetic trees.. Mol Biol Evol.

[40] Storz JF, Beaumont MA, Alberts SC (2002). Genetic evidence for long-term population decline in a savannah-dwelling primate: inferences from a hierarchical bayesian model.. Mol Biol Evol.

[41] Serikawa T, Kuramoto T, Hilbert P, Mori M, Yamada J (1992). Rat gene mapping using PCR-analyzed microsatellites.. Genetics.

[42] Dietrich W, Katz H, Lincoln SE, Shin HS, Friedman J (1992). A genetic map of the mouse suitable for typing intraspecific crosses.. Genetics.

[43] Larkin MA, Blackshields G, Brown NP, Chenna R, McGettigan PA (2007). Clustal W and Clustal X version 2.0.. Bioinformatics.

[44] Guindon S, Gascuel O (2003). A simple, fast, and accurate algorithm to estimate large phylogenies by maximum likelihood.. Syst Biol.

[45] Rodriguez F, Oliver JL, Marin A, Medina JR (1990). The general stochastic model of nucleotide substitution.. J Theor Biol.

[46] Yang Z (1994). Maximum likelihood phylogenetic estimation from DNA sequences with variable rates over sites: approximate methods.. J Mol Evol.

[47] Posada D, Crandall KA (1998). MODELTEST: testing the model of DNA substitution.. Bioinformatics.

[48] Drummond AJ, Ho SY, Phillips MJ, Rambaut A (2006). Relaxed phylogenetics and dating with confidence.. PLoS Biol.

[49] Hasegawa M, Kishino H, Yano T (1985). Dating of the human-ape splitting by a molecular clock of mitochondrial DNA.. J Mol Evol.

[50] Drummond AJ, Rambaut A, Shapiro B, Pybus OG (2005). Bayesian coalescent inference of past population dynamics from molecular sequences.. Mol Biol Evol.

[51] Lukashevich IS, Clegg JC, Sidibe K (1993). Lassa virus activity in Guinea: distribution of human antiviral antibody defined using enzyme-linked immunosorbent assay with recombinant antigen.. J Med Virol.

[52] Demby AH, Inapogui A, Kargbo K, Koninga J, Kourouma K (2001). Lassa fever in Guinea: II. Distribution and prevalence of Lassa virus infection in small mammals.. Vector Borne Zoonotic Dis.

