# Supplementary material for: The Impact of Human Conflict on the Genetics of Mastomys natalensis and Lassa Virus in West Africa
Source: PLoS One. 2012 May 15;7(5):e37068. doi: 10.1371/journal.pone.0037068 (PMC3352846; doi:10.1371/journal.pone.0037068)
Supplement: Table S1 — Dates and locations of animal-trapping sites in Guinea and Ivory Coast. The areas were chosen according to the results published in Lukashevich et al. (1993) [51] and Demby et al. (2001) [52]. They cover representative sites of the principal geographic regions of Guinea where human/rodent presence of Lassa virus was observed. (DOC) [file pone.0037068.s006.doc]

Table S1: Dates and locations of animal-trapping sites in Guinea and Ivory Coast. The areas were chosen according to the results published in Lukashevich et al. (1993) [51] and Demby et al. (2001) [52]. They cover representative sites of the principal geographic regions of Guinea where human/rodent presence of Lassa virus was observed.

* *M. erythroleucus* sample from these localities were not included in the genetic analyses. These stations correspond to regions were both species coexisted in sympatry and they are accordingly indicated on the map.
