# Supplementary material for: The Impact of Human Conflict on the Genetics of Mastomys natalensis and Lassa Virus in West Africa
Source: PLoS One. 2012 May 15;7(5):e37068. doi: 10.1371/journal.pone.0037068 (PMC3352846; doi:10.1371/journal.pone.0037068)

Table S2: Pairs of putative full-sibs and half-sibs identified within stations using Colony 2.0.0.1 [13]. *P* values in the first line correspond to the probabilities of the sibship assignments. *P* values from the two last columns were estimated from resampling procedures implemented in Poptools v3.2.3 [37] and indicate if the LASV positive rats sub-sample in each population are significantly different from the LASV negative *M. natalensis* sub-samples.


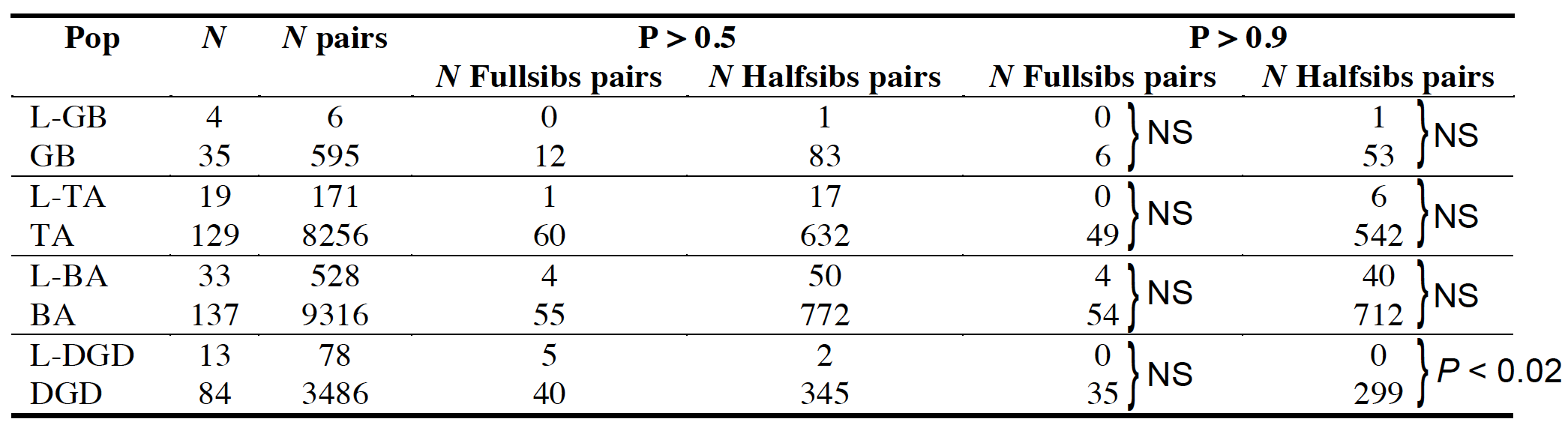

Supplement: Table S2 — Pairs of putative full-sibs and half-sibs identified within stations using Colony 2.0.0.1 [13]. P values in the first line correspond to the probabilities of the sibship assignments. P values from the two last columns were estimated from resampling procedures implemented in Poptools v3.2.3 [37] and indicate if the LASV positive rats sub-sample in each population are significantly different from the LASV negative M. natalensis sub-samples. (DOC) [file pone.0037068.s007.doc]
