# Supplementary material for: The Impact of Human Conflict on the Genetics of Mastomys natalensis and Lassa Virus in West Africa
Source: PLoS One. 2012 May 15;7(5):e37068. doi: 10.1371/journal.pone.0037068 (PMC3352846; doi:10.1371/journal.pone.0037068)
Supplement: Table S3 — Summary statistics for the M. natalensis demographic changes (populations with ≥24 individuals). The estimates were obtained with the algorithm Msvar 1.3. [14] and correspond to effective population sizes and not absolute population sizes. The 95% confidence intervals are provided in the brackets. (DOC) [file pone.0037068.s008.doc]

Table S3: Summary statistics for the *M. natalensis* demographic changes (populations with ≥ 24 individuals). The estimates were obtained with the algorithm Msvar 1.3. [14] and correspond to effective population sizes and not absolute population sizes. The 95% confidence intervals are provided in the brackets.

| Population | Actual population size | Ancestral population size | Time elapsed since bottleneck | Mutation rate |
| --- | --- | --- | --- | --- |
| Bamakama | 8.1  [0.09; 870] | 58,000  [550; 5,200,000] | 25  [0.27; 2,300] | 1.1 10^-3^  [1.3 10^-5^; 0.12] |
| Denguedou | 5.2  [0.07; 620] | 91,000  [890; 7,800,000] | 15  [0.16; 1,300] | 7.9 10^-4^  [9.6 10^-6^; 0.081] |
| Bantou | 5.0  [0.05; 537] | 80,000  [930; 11,000,000] | 14  [0.14; 1,500] | 6.3 10^-4^  [6.2 10^-6^; 0.069] |
| Gbetaya | 7.1  [0.05; 590] | 81,000  [760; 8,900,000] | 19  [0.15; 1,600] | 7.9 10^-4^  [6.3 10^-6^; 0.074] |
| Tanganya | 5.6  [0.05; 690] | 85,000  [650; 8,900,000] | 20  [0.17; 2,000] | 6.6 10^-4^  [6.5 10^-6^; 0.093] |
| Franfina | 8.5  [0.07; 680] | 78,000  [650; 6,800,000] | 23  [0.20; 2,000] | 9.1 10^-4^  [7.9 10^-6^; 0.079] |
| Kalia | 2.9  [0.04; 430] | 85,000  [960; 10,200,000] | 14  [0.14; 1450] | 4.7 10^-4^  [6.3 10^-6^; 0.065] |
