# Supplementary material for: The Impact of Human Conflict on the Genetics of Mastomys natalensis and Lassa Virus in West Africa
Source: PLoS One. 2012 May 15;7(5):e37068. doi: 10.1371/journal.pone.0037068 (PMC3352846; doi:10.1371/journal.pone.0037068)
Supplement: Table S4 — Complete listing of the sequences included in the phylogenetic analysis of the viruses, as identified from databases, or determined in the course of this study. (DOC) [file pone.0037068.s009.doc]

Table S4: Complete listing of the sequences included in the phylogenetic analysis of the viruses, as identified from databases, or determined in the course of this study.

**Acc. Number Name Year Country Region Host**

AF182219 803213 1974 Nigeria Onitsha Human

AF182220 803214 1974 Nigeria Onitsha Human

AF182221 806316 1989 Nigeria Ekpoma Human

AF182222 808031 1994 Nigeria Ekpoma Human

AF182223 806320 1989 Nigeria Aba Human

AF182224 806319 1989 Nigeria Aba Human

AF182225 806321 1989 Nigeria Owerri Human

AF182226 806322 1989 Nigeria Owerri Human

AF182227 803787 1981 Nigeria ? Human

AF182228 806791 1993 Nigeria Jos Human

AF182229 803210 1975 Nigeria Vom Human

AF182230 803212 1976 Nigeria Vom Human

AF182231 807975 1976 Nigeria Vom Human

AF182232 803208 1975 Nigeria Zonkwa Human

AF182233 802662 1980 Sierra Leone Niahun Human

AF182234 801618 1979 Sierra Leone Konia Human

AF182235 803205 1976 Sierra Leone Segbwema Human

AF182236 803209 1975 Sierra Leone Segbwema Human

AF182237 9607290 1996 Sierra Leone Segbwema Human

AF182238 806827 1977 Sierra Leone Gondama Human

AF182239 801105 1978 Sierra Leone Konia Rodent

AF182240 801107 1978 Sierra Leone Konia Rodent

AF182241 801104 1978 Sierra Leone Konia Rodent

AF182242 801106 1978 Sierra Leone Konia Rodent

AF182243 801109 1978 Sierra Leone Konia Rodent

AF182244 801108 1978 Sierra Leone Konia Rodent

AF182245 807875 1982 Sierra Leone ? Rodent

AF182246 807976 1977 Sierra Leone Tongo Human

AF182247 803211 1972 Sierra Leone Panguma Human

AF182248 801101 1978 Sierra Leone Tongo Rodent

AF182249 801103 1978 Sierra Leone Tongo Rodent

AF182250 803206 1976 Sierra Leone Panguma Human

AF182251 801102 1978 Sierra Leone Tongo Rodent

AF182252 9607300 1996 Sierra Leone Tongo Human

AF182253 801100 1978 Sierra Leone Tongo Rodent

AF182254 806843 1993 Sierra Leone Tongo Human

AF182255 9607302 1996 Sierra Leone Tongo Human

AF182256 803972 1982 Sierra Leone Mano Human

AF182257 807974 1976 Sierra Leone Mobai Human

AF182258 803204 1972 Liberia Zorzor Human

AF182259 803201 1972 Liberia Zorzor Human

AF182260 803792 1980 Liberia Zorzor Human

AF182261 806828 1981 Guinea Macenta Human

AF182262 803791 1980 Liberia Zorzor Human

AF182263 803793 1980 Liberia Zorzor Human

AF182264 806829 1981 Liberia Zorzor Human

AF182265 807998 1997 Guinea Nzerekore Human

AF182266 803203 1972 Liberia Zorzor Human

AF182267 807992 1997 Guinea Kissedougou Human

AF182268 807977 1981 Liberia Zorzor Human

AF182269 807868 1996 Guinea Faranah Human

AF182270 808255 1996 Guinea Faranah Rodent

AF182271 803796 1981 Liberia Phebe Human

AF182272 las9608911 1996 Nigeria Jos Human

AF246121 AV 2000 Ivory Coast ? Human

AF333969 CSF 2000 Nigeria Jos Human

AY179173 NL 2000 Sierra Leone ? Human

DQ832667 GB129 2003 Guinea Gbetaya Rodent

DQ832668 GB132 2003 Guinea Gbetaya Rodent

DQ832669 BA263 2003 Guinea Bantou Rodent

DQ832670 BA289 2003 Guinea Bantou Rodent

DQ832671 BA302 2003 Guinea Bantou Rodent

DQ832672 BA350 2003 Guinea Bantou Rodent

DQ832673 BA354 2003 Guinea Bantou Rodent

DQ832674 BA356 2003 Guinea Bantou Rodent

DQ832675 BA366 2003 Guinea Bantou Rodent

DQ832676 BA375 2003 Guinea Bantou Rodent

DQ832677 BA377 2003 Guinea Bantou Rodent

DQ832678 BA378 2003 Guinea Bantou Rodent

DQ832679 BA382 2003 Guinea Bantou Rodent

DQ832680 BA384 2003 Guinea Bantou Rodent

DQ832681 BA686 2004 Guinea Bantou Rodent

DQ832682 TA416 2003 Guinea Tanganya Rodent

DQ832683 TA431 2003 Guinea Tanganya Rodent

DQ832684 TA444 2003 Guinea Tanganya Rodent

DQ832685 TA462 2003 Guinea Tanganya Rodent

DQ832686 TA464 2003 Guinea Tanganya Rodent

DQ832687 TA471 2003 Guinea Tanganya Rodent

DQ832688 TA491 2003 Guinea Tanganya Rodent

DQ832689 TA817 2004 Guinea Tanganya Rodent

DQ832690 TA820 2004 Guinea Tanganya Rodent

DQ832691 TA846 2004 Guinea Tanganya Rodent

DQ832692 DGD4 2005 Guinea Denguédou Rodent

DQ832693 DGD13 2005 Guinea Denguédou Rodent

DQ832694 DGD28 2005 Guinea Denguédou Rodent

DQ832695 DGD35 2005 Guinea Denguédou Rodent

DQ832696 DGD43 2005 Guinea Denguédou Rodent

DQ832697 DGD87 2005 Guinea Denguédou Rodent

DQ832698 DGD104 2005 Guinea Denguédou Rodent

DQ832699 DGD112 2005 Guinea Denguédou Rodent

NC004296 Josiah 1976 Sierra Leone ? Human

LIB1269 2004 Liberia Ganta Human

U8004 LP 1969 Nigeria Lassa Human
