# Supplementary material for: The Impact of Human Conflict on the Genetics of Mastomys natalensis and Lassa Virus in West Africa
Source: PLoS One. 2012 May 15;7(5):e37068. doi: 10.1371/journal.pone.0037068 (PMC3352846; doi:10.1371/journal.pone.0037068)
Supplement: Appendix S1 — Scripts implemented for the spatial analyses. (DOC) [file pone.0037068.s010.doc]

**Scripts implemented for the spatial analyses**

# the following functions need pixmap package installed

#random function needs Rlab package installed

random<-function(x,y,z,dens)

{

img <- read.pnm(x) # reads the ppm image file

red<-round(img@red,2)

blue<-round(img@blue,2)

green<-round(img@green,2)

D<- read.pnm(dens) # reads the density map

density_map <- D@red

newimage<-matrix(0,nrow=length(blue[,1]),length(blue[1,])) # builds the matrix corresponding to the map

for(g in 1:length(blue[,1]))

{

for(h in 1:length(blue[1,]))

{

if(blue[g,h]>=0.90 && green[g,h]<=0.2)

{

newimage[length(blue[,1])-g,h]=1

}

if(red[g,h]>=0.95 && green[g,h]<0.2)

{

newimage[length(blue[,1])-g,h]=2

}

}

}

density_matrix <-matrix(0,nrow=length(density_map[,1]),length(density_map[1,])) # builds the matrix corresponding to the density map

for(g in 1:length(density_map[,1]))

{

for(h in 1:length(density_map[1,]))

{

if(density_map[g,h]>=0)

{

density_matrix[length(blue[,1])-g,h]=density_map[g,h]

}

}

}

newimage<-floor(newimage+0.5)

newimage<-t(newimage)

density_map<-t(density_matrix)

density_map=density_map*density_map

b=newimage==2 # constucts list of refugee camps points (or war zones)

c=length(newimage[b])

a=matrix(0,c,2)

k=0

for (i in 1:length(newimage[,1])) for(j in 1:length(newimage[1,]))

{

if(newimage[i,j]==2)

{

a[k,1]=i

a[k,2]=j

k=k+1

}

}

results<-mat.or.vec(y,z) # matrix of results

distances=mat.or.vec(1,z) # matrix of all possible distances for each points

for(repeats in 1:y) # number of repetitions : y

{

k=1

random <- mat.or.vec(2,z)

random[1,]=sample(1:length(newimage[,1]),z,replace=F) # random lattitude

random[2,]=sample(1:length(newimage[1,]),z,replace=F) # random longitude

f=1

for ( i in 1:z)

{

accept=rbern(1,density_map[random[1,i],random[2,i]]) # accept or not the random generated point according to density

while (newimage[random[1,i],random[2,i]]==1 | accept==0) # check if the generated points is not in the water

{

random[1,i]=sample(1:length(newimage[,1]),1)

random[2,i]=sample(1:length(newimage[1,]),1)

accept=rbern(1,density_map[random[1,i],random[2,i]])

}

newimage[random[1,i],random[2,i]]=3

}

for (i in 1:length(newimage[,1])) for (j in 1:length(newimage[1,])) # to calculate the distances

{

if( newimage[i,j]==3)

{

point=1

for (k in 1:length(a[,1]))

{

distances[point]=sqrt(abs(i-a[k,1])^2+abs(j-a[k,2])^2) # adds all distances to d matrix

point=point+1

}

results[repeats,f]=min(distances) # takes the minimum of each d matrix and adds it to c matrix

f=f+1

}

}

for ( i in 1:z) newimage[random[1,i],random[2,i]]=0 # erases random points for the next repetition

print(repeats)

}

image(newimage) # display the image on screen

return(results)

}

# outbreaks function places outbreak points on given map and calculates distance to conflict area/refugee camp

outbreaks<-function(x,z,w)

{

img <- read.pnm(x)

red<-round(img@red,2)

blue<-round(img@blue,2)

green<-round(img@green,2)

newimage<-matrix(0,nrow=length(blue[,1]),length(blue[1,]))

for(g in 1:length(blue[,1]))

{

for(h in 1:length(blue[1,]))

{

if(blue[g,h]>=0.90 && green[g,h]<=0.2)

{

newimage[length(blue[,1])-g,h]=1

}

if(red[g,h]>=0.95 && green[g,h]<0.2)

{

newimage[length(blue[,1])-g,h]=2

}

}

}

newimage<-floor(newimage+0.5)

newimage<-t(newimage)

points<-read.table(w)

for (i in 1:z)

{

if (points[i,2]<length(newimage[,1]) && points[i,1]<length(newimage[1,])) newimage[points[i,2],points[i,1]]=3

}

image(newimage)

b=newimage==2

c=length(newimage[b])

a=matrix(0,c,2)

k=0

for (i in 1:length(newimage[,1])) for(j in 1:length(newimage[1,]))

{

if(newimage[i,j]==2)

{

a[k,1]=i

a[k,2]=j

k=k+1

}

}

results<-mat.or.vec(1,z)

distances=mat.or.vec(1,z)

k=1

f=1

for (i in 1:length(newimage[,1])) for (j in 1:length(newimage[1,]))

{

if( newimage[i,j]==3)

{

point=1

for (k in 1:length(a[,1]))

{

distances[point]=sqrt(abs(i-a[k,1])^2+abs(j-a[k,2])^2)

point=point+1

}

results[f]=min(distances)

f=f+1

}

}

return(results)

}
